# Effectiveness of stress arousal reappraisal and stress-is-enhancing mindset interventions on task performance outcomes: a meta-analysis of randomized controlled trials

**DOI:** 10.1038/s41598-024-58408-w

**Published:** 2024-04-04

**Authors:** Michel Bosshard, Patrick Gomez

**Affiliations:** 1 Institute for Medical Education, University of Bern, Bern, Switzerland; 2 Graduate School for Health Sciences, University of Bern, Bern, Switzerland; 3 Department of Occupational and Environmental Health, Center of Primary Care and Public Health (Unisanté), University of Lausanne, Lausanne, Switzerland

**Keywords:** Psychology, Human behaviour

## Abstract

Stress arousal reappraisal (SAR) and stress-is-enhancing (SIE) mindset interventions aim to promote a more adaptive stress response by educating individuals about the functionality of stress. As part of this framework, an adaptive stress response is coupled with improved performance on stressful tasks. The goal of this meta-analysis is to evaluate the effectiveness of these interventions on task performance. The literature search yielded 44 effect sizes, and a random-effects model with Knapp-Hartung adjustment was used to pool them. The results revealed an overall small significant improvement in task performance (*d* = 0.23, *p* < 0.001). The effect size was significantly larger for mixed interventions (i.e., SAR/SIE mindset instructions combined with additional content, *k* = 5, *d* = 0.45, *p* = 0.004) than SAR-only interventions (*k* = 33, *d* = 0.22, *p* < 0.001) and SIE mindset-only interventions (*k* = 6, *d* = 0.18, *p* = 0.22) and tended to be larger for public performance tasks than cognitive written tasks (*k* = 14, *d* = 0.34, *p* < 0.001 vs. *k* = 30, *d* = 0.20, *p* = 0.002). Although SAR and SIE mindset interventions are not “silver bullets”, they offer a promising cost-effective low-threshold approach to improve performance across various domains.

## Introduction

Stress is a loyal companion that is omnipresent through all stages of an individual’s life. This complex state is appraised, experienced, and managed in different ways depending on a person’s characteristics and the situational context^1^. Hans Selye, the pioneer in stress research, defined stress as “*the non-specific response of the body to any demand made upon it*”^2^. According to Selye, experiencing stress can yield either positive (eustress) or negative (distress) outcomes. While both sides are present in most major stress theories, the positive aspects have received far less attention in research^3–5^. While prolonged and excessive stress can negatively impact physical and mental health (e.g.^6–8^), short term stress responses evolved to ensure survival and to allow thriving in most demanding situations^6^. The perspective that stress is essential for human functioning, growth, and performance, is not widely spread and for the most part, the term stress has become synonym with distress. This predominantly negative view can be problematic as it indirectly suggests that stress must be avoided or reduced^9^. However, stress is unavoidable in various domains of daily life. Situations that are of personal importance and require an individual to perform (e.g., school examinations, job interviews, sports competitions) will likely trigger stress^9^. Conventional stress management strategies aim at downregulating or ignoring stress altogether^10^. Yet, research suggests that the way an individual perceives stress can lead to differences in the stress response (e.g.^11–14^). Specifically, accepting and appraising stress as functional and helpful instead of harmful, might evoke a more adaptive stress response.

Stress arousal reappraisal (SAR) and stress-is-enhancing (SIE) mindset interventions have the same goal of promoting a more adaptive stress response by educating individuals about the functional aspects of stress^15,16^. They both aim to foster an individual’s agency in channeling and utilizing stress responses in a way that is beneficial for mastering stressors. However, there is a salient difference between these two approaches. Whereas SAR interventions function through situation specific appraisals of perceived task demands and coping resources, SIE mindset interventions frame stress as generally enhancing, independent of how a specific situation is appraised. Acknowledging these similarities and differences, authors have recently proposed integrative models^9,17^.

One line of research has been interested in studying the experiential (e.g., subjective affective experience) and physiological (e.g., cardiovascular activity) effects of SAR and SIE mindset interventions (e.g.^18–20^). A meta-analysis found a small positive effect of both interventions on the subjective stress response but no significant effect on the physiological response^21^. A second line of research has focused on investigating if SAR and SIE mindset interventions can improve performance outcomes (in, e.g., academic examinations^13^, public speaking^11^, sports^14^). The present study aims to analyze this research question meta-analytically.

### Stress arousal reappraisal (SAR) interventions and task performance

SAR (often also called stress reappraisal or arousal reappraisal) refers to the cognitive reframing of the meaning of arousal experienced in the context of stressful situations or tasks^22^. SAR interventions are rooted in the biopsychosocial model of challenge and threat, which frames biological, psychological, and behavioral aspects of stress as interrelated patterns in the context of motivated performance situations (i.e., situations in which an active response is needed to achieve personally relevant goals). According to this model, adaptive cardiovascular and performance enhancing (challenge-oriented) stress responses are achieved when perceived personal resources outweigh perceived situational demands, whereas debilitative (threat-oriented) stress responses are caused by perceiving situational demands as exceeding personal resources^23,24^. SAR interventions advise individuals to reappraise the stress response itself as a resource to cope with the situational demands of the task at hand, thereby causing a shift towards challenge-oriented stress responses. More precisely, they convey the message that the body's response to stressors (i.e., stress arousal) can be functional and adaptive and thus invite individuals to think of their stress arousal as helpful rather than harmful for task performance^17^. For example, Jamieson and colleagues’^25^ SAR interventional material reads as follows: “*People think that feeling anxious while taking a standardized test will make them do poorly on the test. However, recent research suggests that arousal doesn’t hurt performance on these tests and can even help performance… people who feel anxious during a test might actually do better. This means that you shouldn’t feel concerned if you do feel anxious while taking today’s GRE test. If you find yourself feeling anxious, simply remind yourself that your arousal could be helping you do well.*” Revised versions of the SAR intervention were made more exhaustive^26,27^ by clarifying the functionality and evolutionary benefits of the body’s stress response (e.g., an increased heart rate is a sign that the body is being fueled with more oxygen, therefore preparing an individual to perform). A more in-depth analysis of the SAR intervention and the exact mechanism of the biopsychosocial model of challenge and threat can be found elsewhere (e.g.^22^).

To our knowledge, the first study that evaluated the effects of what we consider SAR on performance was conducted by Garcia in 1982^28^. In this study, math-anxious students met with a therapist six times over the course of four weeks. During the meetings, students imagined themselves in a physiological aroused state and were instructed to envision the arousal as sign of the body releasing energy which should be utilized to manage the task ahead. After the training, participants in the SAR condition achieved significantly higher scores on a math exam than participants in the neutral control condition.

More recently, SAR attracted new interest as an intervention capable of improving task performance. Jamieson and colleagues showed that students administered with a SAR intervention consisting of a brief statement (see above) performed significantly better than controls on a math exam but not on a verbal exam^25^. Over the past decade, the effects of SAR interventions on task performance have been evaluated in different domains (academic examinations e.g.^29–31^, tasks in sports^14,32^, and verbal interactions e.g.^11,33,34^).The results appear rather inconsistent, with studies finding SAR interventions to have negative effects on reading span^35^ and math performance^36^, no effects on intelligence tests^37^ and quantitative reasoning^38^, but positive effects on golf putting^14^ and math performance^13^.

### Stress-is-enhancing (SIE) mindset interventions and task performance

Stress mindset theory evolved from the concept of implicit theories^39^, which represent organizing principles that encompass specific beliefs, assumptions, and expectations about a human attribute or construct (e.g., intelligence, personality) and their malleability. A mindset can be described as a cognitive framework that selectively processes information gathered in the environment and can be explicitly altered^40^. Accordingly, stress mindsets refer to beliefs and assumptions that one associates with the nature of stress. Although authors differ to some extend in the conceptualization of stress mindset (e.g.^39,41–43^), a key distinction is between two opposing mindsets: an SIE mindset is represented by beliefs that stress can enhance performance, health, wellbeing, learning, and growth, whereas a stress-is-debilitating (SID) mindset comes with the assumption that stress has debilitating effects. According to stress mindset theory, a person’s mindset guides the anticipation and experience of a stressful event. An SID mindset motivates an individual to avoid or downregulate the stress response and thus its “harmful” consequences. An SIE mindset encourages individuals to accept and utilize the enhancing effects that stress can provide^39,44^. SIE mindset interventions promote the idea that stress mindsets are not fixed and foster positive associations about stress in an attempt to optimize stress responses^39,44^. The goal is for individuals to be able to consciously adopt an SIE mindset when confronted with demanding situations. A more detailed overview of stress mindset theory and its mechanisms is available elsewhere^15,17^.

In an early series of studies, Crum and colleagues provided evidence for correlations between stress mindsets and physiological and behavioral stress responses^39^. Moreover, participants who were shown three 3-min videos that either promoted the enhancing or debilitating effects of stress on performance, health, wellbeing, growth, and learning exhibited corresponding changes in their beliefs about stress, self-reported work performance, and wellbeing (i.e., SIE mindset increased performance and wellbeing)^39^. Following this initial effort, a shorter 3-min version focused on cognitive performance^12^ and an extensive (60–120 min) version of the SIE mindset intervention were introduced^45^. In general, the different adaptations of the SIE mindset intervention were consistent in promoting a more positive stress mindset, with the extensive version evoking the most sustainable changes in stress mindset^45^. Despite changes in people’s stress mindset have been consistently reported, the effects of the stress mindset interventions on performance have not been as clear. One study found positive effects on articulation rate of tongue twisters^46^, while performance improvements in other studies were dependent on factors such as genotype (Stroop-task^47^) or feedback condition (alternative use task^12^). Further, performance enhancing effects in academics were only achieved by combining SIE mindset interventions with other content, such as imagery exercise^48^ or growth mindset about abilities/intelligence^49^. The intervention failed to improve academic grades in a study with disadvantaged students^50^.

### Present review

We bring forward two concerns that might prevent optimal use and development of the SAR and SIE mindset interventions. First, research has reported inconsistent results about the effects of these interventions on performance outcomes. This makes it difficult to predict their future effectiveness when planning an intervention. It might also be a reason why the interventions went through multiple iterations and combinations with other elements, which leads to the second concern. The studies about both interventions possess a high degree of heterogeneity in terms of, e.g., intervention content and performance task. These differences introduce additional difficulties for researchers and educators to make informed decisions. We conducted this meta-analysis to address the following research questions: (1) How effective are SAR and SIE mindset interventions in improving task performance? (Overall effect) (2) Does the effectiveness of the interventions depend on specific studies’ characteristics (type of intervention, type of control group, type of task, reflection exercises, gender ratio).

## Methods

The current meta-analysis is based on the PRISMA 2020 checklist (Supplementary Table S1) and preregistered on OSF Registries: https://osf.io/45w6h.

### Inclusion and exclusion criteria

The following inclusion criteria were derived from the PICOS^51^ framework. We included (1) randomized controlled trials (RCTs) with a between-subjects design (2) in English, German, French, Italian, or Spanish, in which (3) either an SAR or SIE mindset intervention was applied (4) to target an active stress-inducing task, (5) for which performance was objectively assessed or rated in a standardized way, and (6) was compared to a neutral or active (stress related) control condition. We excluded (1) studies not meeting all inclusion criteria, (2) review articles, (3) duplicate articles or overlapping data, and (4) studies from which data could not be reliably extracted and for which we did not receive the necessary information from the authors upon request. The health of the participants was not a criterion (i.e., studies with both clinical and non-clinical populations were accepted).

### Study selection

#### Standardized search

To formulate a suitable search string for the databases, we initially conducted a forward search on articles that are omnipresent in the SAR and stress mindset literature. After identifying 20 relevant articles, we analyzed the vocabulary that was used to describe the two interventions. We then combined related terms for *stress* (i.e., arousal, anxiety) and *reappraisal* (i.e., reframing, reinterpretation) as well as *stress mindset* to a single search string. By adding a proximity parameter, irrelevant articles were filtered out (e.g., stress and reappraisal could only be separated by a maximum of two words). Articles had to include respective terms in either the title, abstract, or keywords. To minimize the risk of missing out on potentially relevant articles, we chose a sensitive approach by not including performance (outcome) in the search string. Database specific strings can be found in the Supplementary Table S2.

The standardized article search was conducted on December 12, 2022. We searched the following databases to cover various research fields, peer-reviewed articles, dissertations, and theses: Psycinfo, Scopus, Web of Science, MEDLINE, ERIC, ProQuest Dissertations & Theses, and Cochrane.

The two authors independently assessed the articles based on the eligibility criteria. Initially, both authors screened the title and abstract of all articles and decided for which articles the full text should be retrieved. When no consensus could be reached between the reviewers, an inclusive approach was chosen.

#### Exploratory search

After the electronic database search, we further conducted an exploratory backward and forward reference search based on the most cited and recent articles and contacted authors who have published relevant articles and asked them for unpublished literature.

### Data collection process

A data extraction sheet was pilot tested on 10 randomly selected studies. The purpose of the extraction sheet was to collect necessary statistics for effect size calculation (e.g., means, *SD*/*SE*, group size) or parameters that could be transformed into the desired effect size (e.g., *F*-values from univariate analyses, *p*-values). When effect sizes could not be reliably derived because of missing data, the authors of the articles were contacted by email. If the authors did not reply within two months, a second attempt was made to retrieve the necessary data.

Another goal was to code study characteristics for subgroup analysis, which were further refined during the data collection process. Therefore, we extracted information regarding study design, content of the intervention and control groups, type of outcome, and participant specifics (e.g., gender). Although the overall quality of the studies was high because we only included RCTs, we additionally assessed the quality of each study on four items (see Supplementary Note 1). The items were based on the Consolidated Standards of Reporting Trials for Social and Psychological Interventions (CONSORT-SPI 2018^52^) and aspects that were not already given by RCTs (e.g., randomization). Each study received a quality score ranging from 0 to 4 (see Supplementary Table S3 for the individual ratings). During the data collection process, all outcome data was extracted in duplicate by the two authors. For the coding of additional study characteristics, one reviewer was tasked with extracting the data from the studies, while the second reviewer double-checked the extracted characteristics. There was no disagreement between reviewers. Table 1 summarizes the studies’ core characteristics.

**Table 1:** Studies’ characteristics*.*

Study	N. *d*’s	*N*	Mean age	Sex % female	Quality score	Intervention	Reflection exercise	Control group(s)	Task
Akinola et al.^11^	1	97	24.0	58.8	4	SAR	No	Neutral	Salary negotiation (P)
Baynard-Montague & James^46^	1	60	22.8	78.3	4	SIE	No	Active	Tongue twisters (P)
Beltzer et al.^53^	1	82	25.0	65.9	4	SAR	Yes	Neutral	TSST speech (P)
Brady et al.^29^	2	431	NA	58.0	3	SAR	No	Neutral	Exam (C)
Chalmers^54^	2	78	NA	69.5	2	SAR	No	Neutral / Active	Math & karaoke (P)
Crum et al.^12^	2	113	24.1	65.3	4	SIE	Yes	Active	Alternative use task (C)
Crum et al.^47^	1	106	24.1	65.4	4	SIE	Yes	Active	Stroop task (C)
Erazo^55^	1	62	21.6	77.4	2	SAR	Yes	Active	TSST math (P)
Ganley et al.^38^	1	116	19.7	66.0	3	SAR	No	Neutral	Quantitative reasoning (C)
Garcia^28^	2	31	NA	100	1	SAR & imagery	Yes	Neutral	Math exam (C)
Goyer et al.^50^	2	98	NA	67.9	3	SIE	Yes	Active	Final week exams (C)
Griffin & Howard^56^	1	135	19.5	69.9	4	SAR	No	Neutral	Socio-evaluative speech (P)
Gurera & Isaacowitz^57^	1	120	41.0	51.6	3	SAR	No	Active	Subtraction (C)
Hangen et al.^58^	2	204	19.9	75.8	4	SAR	Yes	Neutral / Active	Math competition (C)
Jacobs^36^	1	232	22.6	46.0	2	SAR	Yes	Neutral	Math exam (C)
Jacquart et al.^59^	2	76	21.8	70.1	3	SAR & physical exercise	Yes	Neutral	TSST math (P)
Jamieson et al.^25^	1	28	NA	42.9	3	SAR	No	Neutral	Math exam (C)
Jamieson et al.^27^	1	81	29.4	68.8	4	SAR	Yes	Active	Math exam (C)
Jamieson et al.^13^	1	339	24.9	62.2	4	SAR	Yes	Active	Math exam (C)
John-Henderson et al.^30^	1	97	20.9	100	3	SAR	No	Neutral	Math exam (C)
Johns et al.^35^ (Study 3)	1	58	NA	100	3	SAR	No	Neutral	Math exam (C)
Johns et al.^35^ (Study 4)	2	75	NA	61.7	3	SAR	No	Neutral	Reading span task (C)
Keech et al.^48^	1	109	19.1	62.7	4	SIE & imagery	Yes	Neutral	GPA (C)
Mesghina et al.^60^	1	284	NA	79.4	4	SAR	Yes	Neutral	Neuroscience learning (C)
Mesghina et al.^61^ (Study 1)	1	97	NA	61.4	4	SAR	Yes	Neutral	Working memory & reasoning (C)
Mesghina et al.^61^ (Study 2)	1	149	NA	77.4	4	SAR	Yes	Neutral	Working memory & reasoning (C)
Moore et al.^14^	1	50	20.2	44.0	3	SAR	Yes	Neutral	Golf putting task (P)
Ott^37^	1	496	20.9	68.8	3	SAR	No	Neutral	Intelligence test (C)
Oveis et al.^33^	2	130	20.9	59.8	4	SAR	No	Active	Product pitch (P)
Reza et al.^62^	1	664	NA	57.9	3	SAR	Yes / No	Neutral	Programming exam (C)
Rozek et al.^31^	1	285	NA	53.0	3	SAR	Yes	Active	Biology exam (C)
Sammy et al.^32^	1	54	21.7	38.9	4	SAR	No	Neutral	Dart throwing task (P)
Taber^63^	1	268	23.1	66.8	3	SAR	Yes	Neutral	Math exam (C)
Yeager et al.^49^	1	119	NA	NA	3	SAR/SIE & growth mindset	Yes	Neutral	Exams (C)
Zhu^34^	1	95	20.4	27.4	2	SAR	Yes	Active	Business pitch (P)

*SAR* stands for stress arousal reappraisal intervention, *SIE* stands for stress-is-enhancing mindset intervention, *P* stands for public performance task, *C* stands for cognitive written task, *TSST* stands for Trier Social Stress Test, *GPA* stands for grade point average.

### Effect size calculation

To evaluate how SAR and SIE mindset interventions affect performance compared to control conditions, we used Cohen’s *d*^64^ for the main analysis. Because the studies were performed in various domains (e.g., academic, sports), the study designs and performance outcomes were highly heterogeneous. Consequently, we assumed a random-effects model for the analysis. Since the performance outcome in all studies was continuous, we applied restricted maximum likelihood estimator to evaluate the variance of the heterogeneity τ^2^ ^65^. Further, the Knapp-Hartung adjustment was used in the estimation of the confidence interval of the pooled effect size to minimize the probability of a false positive result^66^. We used a generic inverse variance approach to weigh the individual effect sizes. Some of the effect sizes had to be reversed since smaller outcome values indicated better performances^14,32,46,47,59^. A forest plot was used to illustrate the pooled effect size and individual effect sizes. For the main analysis, R (version 4.3.3^67^) package meta^68^ was used.

Whenever possible, we extracted the raw data including mean, *SD*, and sample size (*n*) of the intervention and control group to calculate the effect size and *SE* of each study. In one study^50^, the *SE* had to be extracted from the displayed barplot. For studies where the raw data was missing, we converted the available statistics (e.g., *F*-values from univariate analysis, *p*-values) into the desired effect size and *SE*. If the group sizes were missing and not provided by the authors upon request, we assumed equal group sizes. When performance was measured multiple times (e.g., baseline, immediately after intervention, days later), we included and averaged all outcomes after the intervention. We disregarded pre-test data and change scores for the analysis because this data was not consistently reported, and mixing standardized outcomes for change scores and single measures was not sensible^69^. Possible baseline differences should have occurred randomly and therefore balanced out across studies. If two or more performance outcomes were reported, and none was declared or could be identified as the main outcome, we aggregated effect sizes and *SE*s for all outcomes. We additionally conducted a three-level meta-analysis and a meta-analysis with robust variance estimation and sensitivity analysis for the assumed correlation ρ (using R-package metafor^70^ and clubSandwich^71^) to account for dependencies caused by effect sizes from the same study (see Supplementary Tables S4 and S5). All three approaches led to identical results. For simplicity’s sake, we chose to report aggregated effect sizes. When studies reported independent results for multiple subgroups (e.g., first year students, upper year students^29^), we treated the result for each subgroup as an individual study. For studies that compared SAR or SIE mindset interventions to more than one other condition, we included all relevant comparisons and reported them as individual studies. Relevant comparisons were either neutral control conditions unrelated to stress (e.g., no information, summary about brain) or active control conditions that discussed stress but did not portray it as performance enhancing (e.g., SID mindset, ignore stress). If this meant that the same group of participants was used in more than one comparison (e.g., SAR vs. neutral control, SAR vs. active control), the respective group size was adjusted in the calculation of the effect size and *SE* (e.g., halved for two comparisons). Other types of experimental/control conditions were rare and were not considered in our analyses. For example, Ganley et al.^38^ tested five groups: SAR, “*excited*”, expressive writing, “*look ahead*”, and a neutral control group. We only included the comparison between the SAR group and the neutral control group. No study was excluded in this process.

### Publication bias assessment

A visual inspection of funnel plots, the trim and fill method^72^ (R-package meta^68^), and Egger’s test^73^ (R-package tidyverse^74^) were conducted to evaluate possible asymmetry in effect sizes. A *p*-curve analysis (R-package dmetar^75^) additionally indicated whether an evidential value was present or if the data emerged from selective reporting or *p-*hacking.

### Subgroup analysis

As per pre-registration, we sought to analyze the moderating effects of 11 factors. Due to missing data, lack of variance, or the impossibility to create meaningful subgroups, the following factors could not be considered: delivery of intervention, timing of intervention, age of participants, setting, population, and expertise on task. The following aspects were considered for subgroup analyses.

#### Type of intervention

A first central distinction was made between SAR and SIE mindset interventions. A third category included all “mixed” interventions, which could include additional elements (e.g., SAR and imagery exercises^28,48^).

#### Control group

We made a distinction between neutral control groups that were unrelated to stress, and active control groups that altered the perception of stress (e.g., SID mindset, ignore stress).

#### Type of task

Two types of tasks were defined to group the individual studies: cognitive written tasks and public performance tasks. Cognitive written tasks included academic examinations and exercises (e.g.^25,31,57^), intelligence tests^37^, Stroop tasks^47^, alternative uses tasks (creativity)^12^ and working memory tasks^61^. Importantly, participants working alone on these tasks were not directly evaluated or judged by others. Public performance tasks were defined as tasks during which participants performed in front of an audience or interacted verbally with another person. These included the Trier Social Stress Test^53,55,59^, salary negotiations^11^, tongue twisters^46^, business pitches^33,34^, a golf putting task^14^, and a dart throwing task^32^. Social evaluation, which is a key element of public performance tasks, is a major stress factor^76^. Therefore, it is important to consider possible moderating effects caused by the nature of the task.

#### Reflection exercises

Reflection exercises instructed participants to answer questions, summarize the core information of SAR/SIE mindset interventions, reframe past stressful experiences, or envision how stress can be helpful in future scenarios (e.g., “*In your own words please briefly describe how this information can help you perform well on your exam today*”^27^). These reflection exercises had the goal to further help the participants internalize the idea that stress/arousal can enhance performance. Therefore, we differentiated between studies that included reflection exercises and those that did not.

#### Gender ratio

Male and female participants might be differently receptive to the two interventions. Not only do men and women experience stress differently but they also show different coping patterns^77,78^. There is evidence that gender modulates the effectiveness of different types of emotion regulation instructions and specifically SAR instructions on psychophysiological and performance outcomes^58,79^. Thus, we tested gender ratio as a potential moderator.

## Results

### Study selection

**Figure 1:**
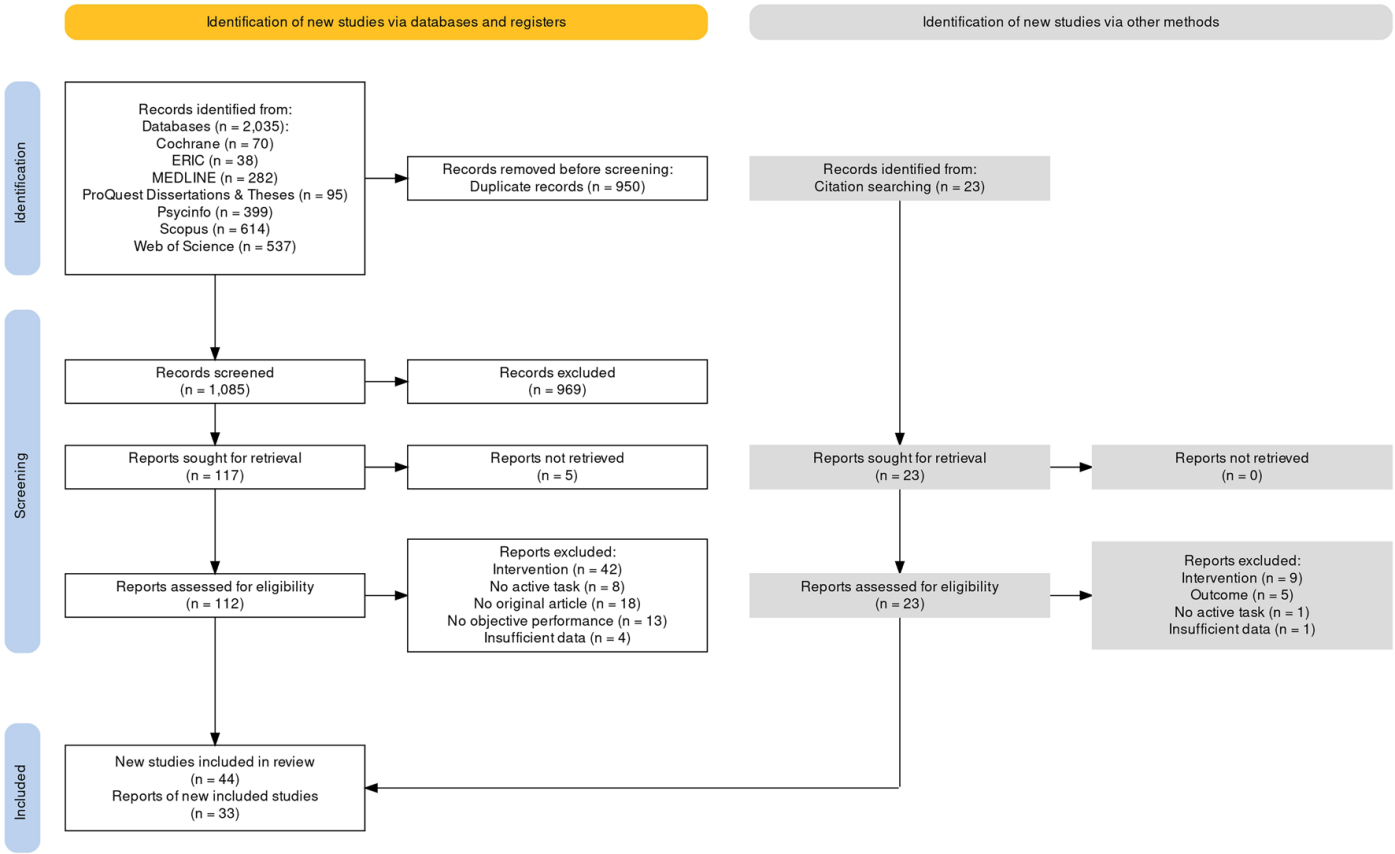
PRISMA 2020 flow diagram.

The standardized literature search yielded 2035 results, of which 1085 remained after removing duplicates. The agreement between reviewers on title and abstract screening was on a moderate level with a Cohen’s Kappa of 0.52. Articles were only excluded if both raters agreed on exclusion. This resulted in the inclusion of 117 articles, for which 112 full texts could be retrieved and reviewed in a second step. Three of the missing articles were study registrations for which the full text was not published yet, and two articles could not be accessed and were not provided by the authors upon request. Five articles were excluded due to insufficient statistics^80–84^. The exploratory backward and forward reference search provided another 23 full texts that were included for screening. Of the 135 full texts analyzed by the two reviewers, 33 were included in the meta-analysis. Lastly, 13 researchers were contacted for additional unpublished data but did not provide any. The individual steps of the study selection procedure are illustrated in the PRISMA flow diagram (Fig. 1).

### Study characteristics

In the end, 35 studies (33 articles) provided 44 effect sizes of which 35 were from peer reviewed publications and 9 from non-peer reviewed dissertations and theses (see Supplementary Note 2 for effect size aggregation). A total of *N* = 5638 participants were included in this meta-analysis of which 65.1% were female (weighted mean). None of the studies investigated a clinical population. The weighted mean age of the participants was 22.6 years. The quality of the studies ranged from 1 to 4 points, with a mean of *M* = 3.26 (*SD* = 0.79). Further study characteristics are reported in the subgroup analysis and in Table 1.

### Overall effect

**Figure 2:**
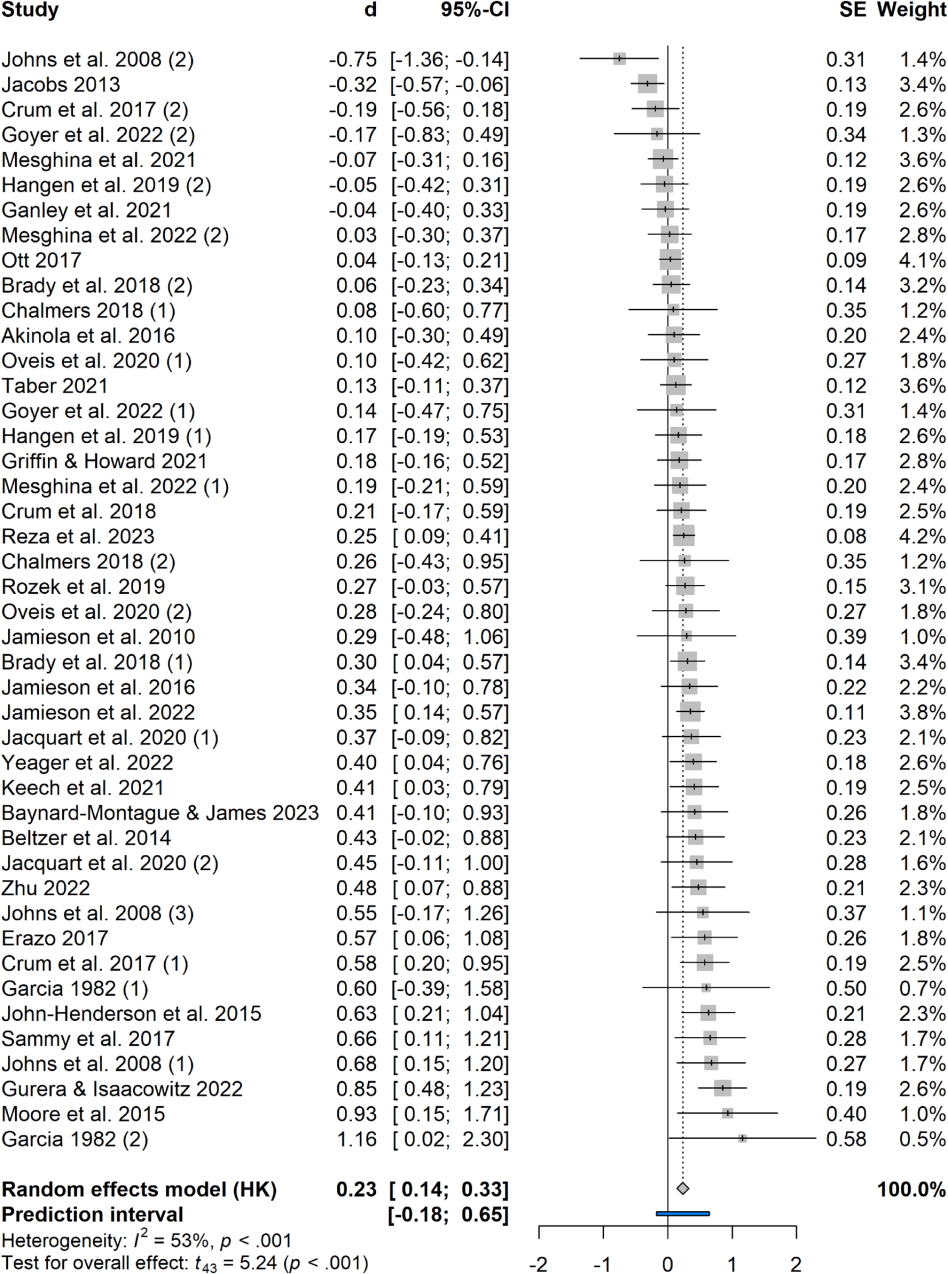
Forest plot. Different effect sizes from the same article were numbered accordingly (1), (2), (3).

The pooled effect size of *d* = 0.23, 95% CI [0.14, 0.33], *p* < 0.001, indicated a small significant effect of the interventions on performance outcomes. Significant heterogeneity was indicated by a τ^2^ = 0.04, 95% CI [0.01, 0.10] and was estimated at a moderate level by an *I*^2^ = 52.6%, 95% CI [33.2%, 66.4%]. The prediction interval [− 0.18, 0.65] suggested that future studies might find small negative effects to moderate positive effects. The individual and pooled effect sizes are displayed in the forest plot (Fig. 2).

### Subgroup analysis

#### Type of intervention

Most studies investigated SAR interventions (*k* = 33). Six studies evaluated SIE mindset interventions, and five studies evaluated SAR or SIE mindset interventions that were supported by additional content (mixed interventions). The effect sizes of the three subgroups were significantly different from one another (*Q*(2) = 7.08, *p* = 0.029). Mixed interventions achieved the largest effect (*d* = 0.45, 95% CI [0.24, 0.66], *p* = 0.004), followed by SAR interventions (*d* = 0.22, 95% CI [0.11, 0.32], *p* < 0.001) and SIE mindset interventions (*d* = 0.18, 95% CI [− 0.15, 0.51], *p* = 0.22). Heterogeneity remained on a moderate level within the subgroups for SAR interventions (*I*^2^ = 56.8%) and SIE mindset interventions (*I*^2^ = 50.1%). The mixed interventions group yielded no between-study heterogeneity (*I*^2^ = 0.0%).

#### Control group

Most studies included a neutral control condition (*k* = 30), whereas the remaining ones used an active stress related control condition (*k* = 14). The between group difference was not significant (*Q*(1) = 1.47, *p* = 0.23). Notably, a small significant (*d* = 0.20, 95% CI [0.09, 0.31], *p* = 0.001) effect size resulted from studies using a neutral control condition. The effect size was larger for studies using an active control condition (*d* = 0.31, 95% CI [0.14, 0.48], *p* = 0.001). Heterogeneity within the subgroups was similar to the overall heterogeneity (neutral, *I*^2^ = 50.9% vs. active, *I*^2^ = 48.2%).

#### Type of task

About two thirds of the tasks were cognitive written tasks (*k* = 30), and one third were public performance tasks (*k* = 14). The results of the subgroup analysis suggest a trending difference between these two task types (*Q*(1) = 3.43, *p* = 0.064). The effect size was smaller for cognitive written tasks than public performance tasks (*d* = 0.20, 95% CI [0.08, 0.31], *p* = 0.002 vs. *d* = 0.34, 95% CI [0.22, 0.46], *p* < 0.001). For cognitive written tasks, the heterogeneity was at a moderate level (*I*^2^ = 62.0%), whereas there was no between-study heterogeneity for public performance tasks (*I*^2^ = 0.0%).

#### Reflection exercises

Twenty-six studies instructed participants to reflect on the information that was presented about stress/arousal, whereas 17 did not include this element. One study alternated between self-reflection and no self-reflection and was therefore removed from this analysis^62^. There were no significant differences in the effect sizes of these two approaches (*Q*(1) = 0.07, *p* = 0.80; reflection exercises *d* = 0.23, 95% CI [0.11, 0.34], *p* < 0.001; no reflection exercises *d* = 0.25, 95% CI [0.07, 0.44], *p* = 0.011). Between-study heterogeneity was slightly lower for tasks with (*I*^2^ = 51.2%) than without (*I*^2^ = 59.1%) reflection exercises.

#### Gender ratio

Gender ratio had no significant impact on the effect size (β = 0.007, 95% CI [0.10, 0.12], *p* = 0.89) and did not explain any between-study heterogeneity (*I*^2^ = 55.9%). For this analysis, a square root transformation of gender ratio was used to achieve a normal distribution.

#### Quality/risk of bias

The meta-regression suggested that quality was not a significant predictor of the effect size (β = − 0.057, 95% CI [− 0.18, 0.07], *p* = 0.38). Accordingly, the unexplained heterogeneity in effect sizes remained similar at *I*^2^ = 54.8%.

### Publication bias

**Figure 3:**
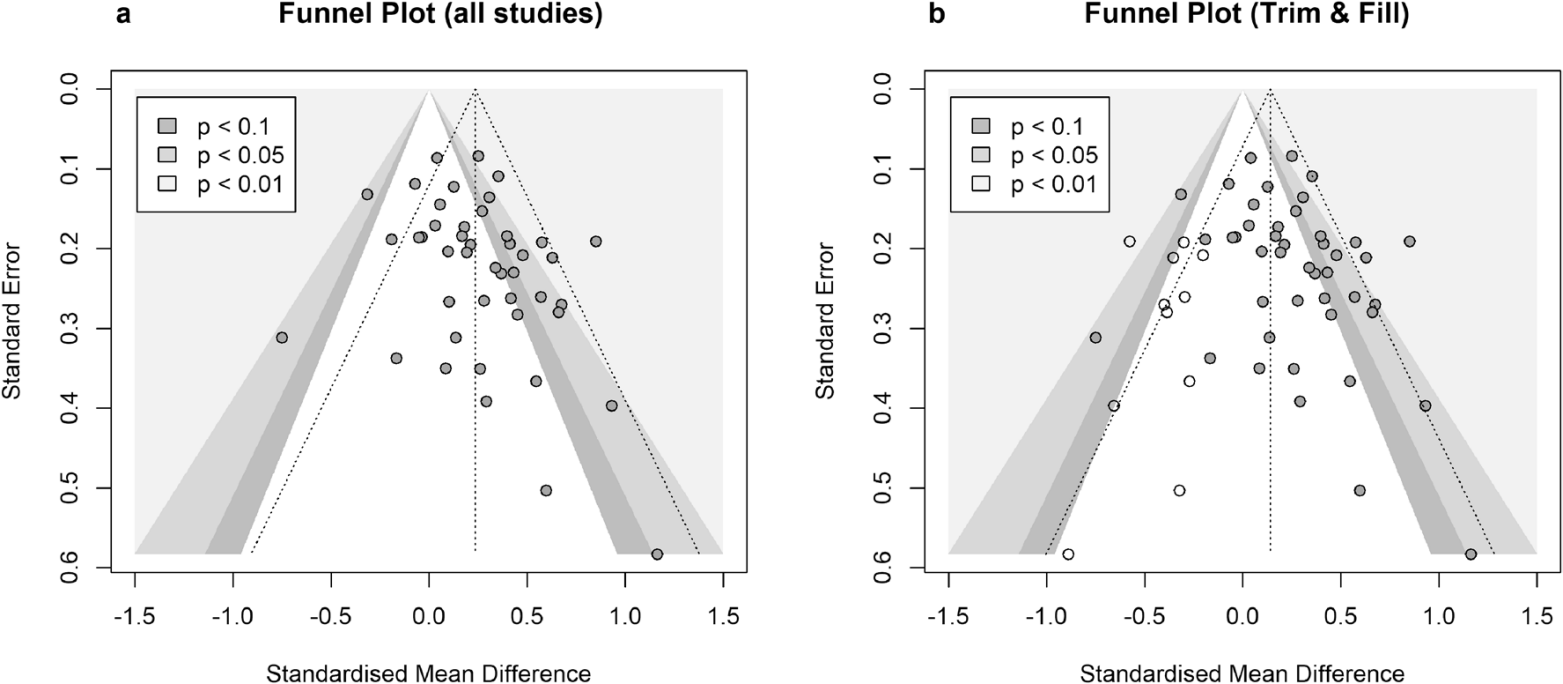
Funnel plot of the effect sizes before (**a**) and after (**b**) trim and fill method has been applied.

Several approaches were applied to investigate the possibility of publication bias. First, by inspecting the funnel plot (Fig. 3a) an asymmetry in effect sizes became visible. Smaller studies (i.e., higher *SE*s) had a disproportionate amount of effect sizes larger than the pooled effect size. The trim and fill method^72^ suggested that 11 studies with a smaller effect size than the pooled effect were missing (indicated by white dots in Fig. 3b). After adding the missing studies to the analysis, the pooled effect size decreased to *d* = 0.14 (95% CI [0.04, 0.24]), yet remained significant (*p* = 0.007). A significant Egger’s test further endorsed the assumption of asymmetry in the effect sizes (*β*_0_ = 1.09, 95% CI [0.07, 2.11], *t* = 2.10, *p* = 0.042).

**Figure 4:**
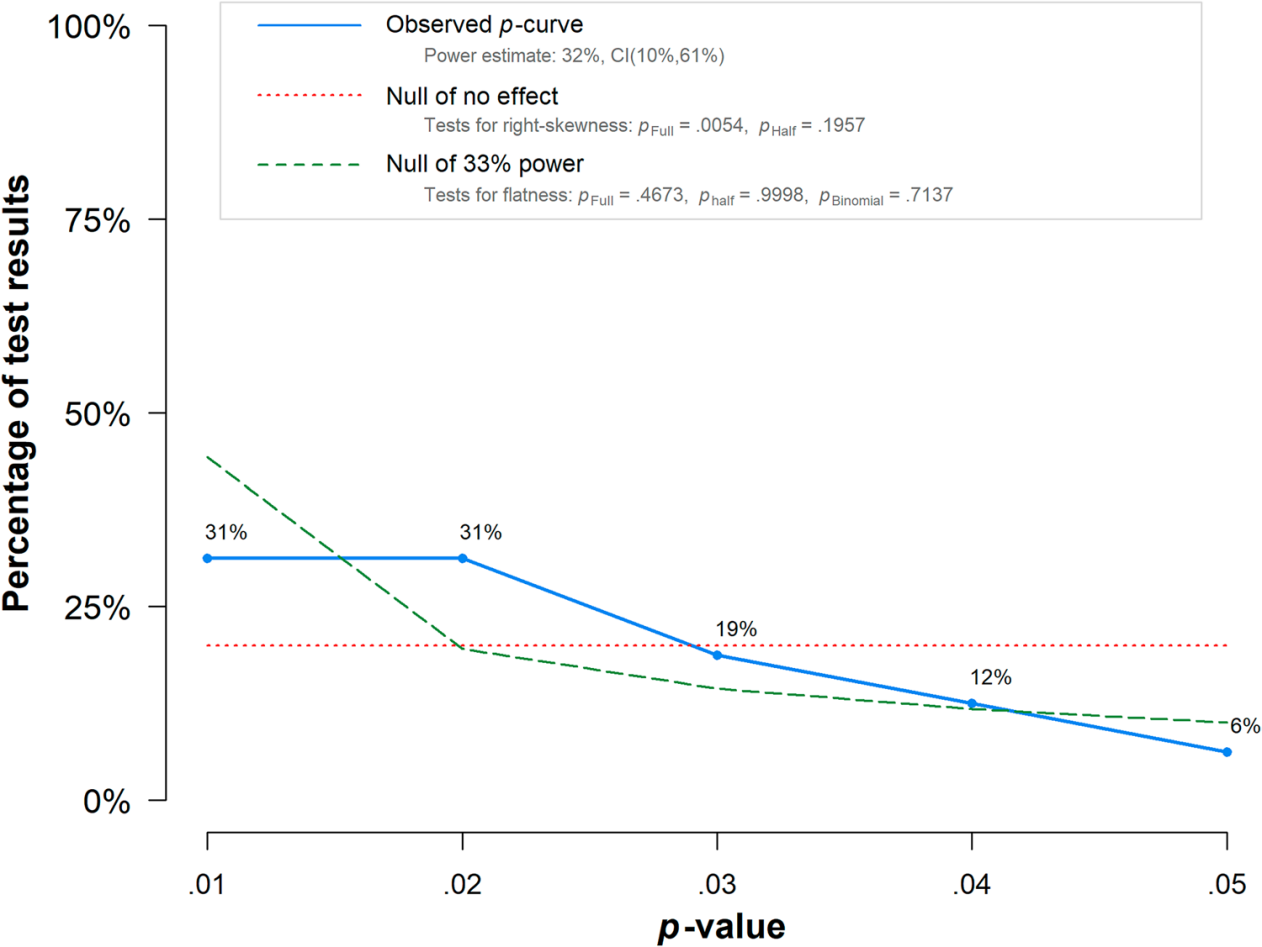
*P*-curve illustrating the distribution of significant *p*-values. The observed *p-*curve includes 16 statistically significant (*p* < 0.05) results, of which 12 are *p* < 0.025.

Finally, we utilized *p*-curve method to analyze the distribution of *p*-values^85,86^. As shown in Fig. 4, there was no indication for the absence of evidential value because both tests for flatness were not significant (*p*_Full_ = 0.47, *p*_Half_ = 1). However, the *p*-curve neither supported the presence of an evidential value because only one test for right-skewness was below 0.1 (*p*_Full_ = 0.005, *p*_Half_ = 0.20).

## Discussion

### Overall effect of the interventions on task performance

In this meta-analysis, we evaluated the effectiveness of SAR and SIE mindset interventions on performance outcomes. We summarized the results of 44 effect sizes retrieved from 35 studies conducted in varying domains, covering cognitive written (e.g., academic exams) and public performance tasks (i.e., performing in front of an audience). The pooled effect size revealed a small positive effect of the interventions on task performance. The differences in studies’ characteristics came with a moderate degree of between-study heterogeneity. We tried to resolve this heterogeneity by conducting subgroup analyses.

### Moderating effects of studies’ characteristics

The type of intervention was the only factor that was found to significantly moderate the effect of the interventions. Interventions that complemented SAR or SIE mindset instructions with additional content (categorized as mixed interventions) achieved the largest effect size (*d* = 0.45). The mixed interventions group itself contained varying elements. An exact mechanism can therefore not be specified. However, as argued by Keech and colleagues^48^, the supportive elements may facilitate proactive coping behavior in general, possibly because the responsible neural networks are stimulated more thoroughly and readily accessible in stressful situations where cognitive capacities are limited^87^. However, compared to SAR-only and SIE mindset-only interventions, mixed interventions cannot be interpreted as easily due to possible confounding effects. While most recent research is already tending towards mixed approaches (e.g.^48,49,59,88^), these are not yet clearly defined and still offer possibilities to explore and experiment with.

Both SAR interventions and SIE mindset interventions produced a small increase in task performance (*d* = 0.22 and *d* = 0.18, respectively), with only the former type of intervention being statistically significant. In terms of effect size, the two interventions do no differ significantly, however, the small number of studies on SIE mindset interventions (*k* = 6) leads to a large estimate uncertainty which makes it difficult to interpret the results. Research comparing SAR and SIE mindset interventions is necessary to determine if and under what conditions these two approaches differ in their effectiveness on task performance.

The subgroup analysis also revealed a trend suggesting that SAR and SIE mindset interventions are more beneficial for public performance tasks than for cognitive written tasks. Social evaluation, which characterizes public performance tasks, induces particularly high physiological stress as indexed by cortisol level^76^. A possible explanation for this task related difference is that individuals in the control groups were better at coping with the lower stress level of cognitive written tasks than with the higher stress level of public performance tasks. However, there is also research indicating that reappraisal of high stress levels is difficult, and other strategies such as distraction are preferred^89,90^. Another aspect that might play a role in explaining the observed task related difference is the higher physical/bodily involvement in public performance tasks than in cognitive written tasks. One could speculate that the bodily signs of arousal are more relevant in public performance tasks and, thus, reframing them as helpful may be more meaningful and useful for performance enhancement.

The type of control condition (neutral vs. active) was not a significant modulator of the effect size, although the estimated effect size for the comparison with neutral control conditions was smaller than for the comparison with active control conditions (*d* = 0.20 vs. *d* = 0.31). This small difference in effect size appears plausible given that the active control conditions mostly tended to induce a threat state or SID mindset, which are supposed to be detrimental for performance outcomes (e.g.^58,75^). It is essential that the interventions yielded a significant effect when compared to a neutral control condition, as this isolates the positive effect of the SAR and SIE mindset interventions on task performance.

The addition of reflection exercises to the interventions offers two functions. First, it should help participants endorse the messages of the interventions and second, it provides an easy way to evaluate if participants paid attention to the presented information^27^. While these exercises fulfill the latter goal, the present meta-analysis suggests that they are redundant when it comes to improving task performance.

While one study found significant gender differences in the effect of a SAR intervention on task performance^58^, the gender effect does not appear when considering all published studies. Hangen and colleagues^58^ attributed the observed gender effect in their study to the unique competitive environment of the math task.

Finally, the quality of the studies was on average high and did not significantly moderate the effect of the interventions.

### Publication bias

Concerns regarding publication bias must be addressed since smaller studies resulted in proportionally larger effect sizes. The result remained significant after applying the trim and fill method to compensate for possibly missing studies, still, the drop in effect size from *d* = 0.23 to *d* = 0.14 is worth noting. However, the *p-*curve analysis could neither support the presence nor absence of an evidential value. This indicates that more evidence is necessary to confirm a true effect.

### Limitations and strengths

Several limitations should be mentioned to put the current findings into perspective. First, most of the studies investigated rather short-term effects. Thus, it remains unclear if the interventions potentially have long lasting effects on task performance. Nevertheless, studies that investigated long-term effects demonstrated positive lasting effects for several months (e.g.^13,25,27,29,48,49,62^ but see^50^). What remains unclear however is if these effects are also transferable to unrelated situations (e.g., from academics to sports). More research investigating the duration and transferability of the performance effects would be a valuable addition. On top of this, we were unable to weigh in the time that had passed between intervention and task, since it was often not possible to accurately identify this information.

Another concern is that most studies did not include baseline performance measures. Therefore, we had to focus on between group comparisons, and possible baseline differences could not directly be accounted for. By including 35 studies in the meta-analysis, random baseline differences should balance each other out. Nevertheless, it is worth noting that in at least one case^32^, the meta-analysis yielded a significant effect size, although the study reported a nonsignificant effect when controlling for the baseline performance in the analysis. We conclude that there is a need for pre-post longitudinal studies testing changes over time in performance outcomes following SAR and SIE mindset interventions.

Further, the positive effects of SAR and SIE mindset interventions on performance must be treated with caution since the performance outcome was not one single standardized measure. Although the subgroup analysis about the effect of the type of task combined tasks with similar characteristics and therefore provided some additional insights, the studies belonging to the same group were still heterogeneous. On the positive side, this diversity in the type of task illustrates that the effects of the interventions are not specific to a single task but rather universally applicable.

Lastly, the subgroup analysis included comparisons of subgroups that were unbalanced or small in terms of number of studies. In particular, 33 studies examined SAR interventions but only 6 studies SIE mindset interventions. Therefore, more studies are needed for a well-grounded estimation of the effect size for SIE mindset interventions. In the end, we were unable to explain between-study heterogeneity completely with the conducted subgroup analysis. Because the studies within subgroups were inconsistent (heterogeneity did not get resolved), it is difficult to draw nuanced conclusions.

A strength of the current meta-analysis is the selection of studies. We only included RCTs, which meant that the quality of the studies was largely high. We further chose a sensitive literature search approach, conducted a forward and backward search, and contacted many authors. By doing so, we retrieved gray literature and included a substantial number of dissertations to provide a comprehensive picture of the literature on the performance effects of SAR and SIE mindset interventions.

## Conclusion

In conclusion, this meta-analysis has shown that SAR and SIE mindset interventions are performance-enhancing across varying domains. Therefore, these interventions can also be considered promising for novel tasks that have not yet been researched. Because these interventions are self-administered, brief, low-threshold, and low-cost, they have the potential to be disseminated to many people, including those facing barriers. However, it must be noted that the overall effect size is small, and therefore these interventions should not be perceived as “silver bullets” for improving task performance. The interventions have proven especially effective for public performance tasks and when their content was supported by additional elements. Investigating the type of additional information that best complements SAR and SIE mindset interventions and the exact mechanism of action of these mixed approaches are important avenues for future research.

### Supplementary Information

Supplementary Information.

## Data Availability

Data supporting the findings of this study are available in the tables and figures of this article. The datasets generated and analyzed during the current study are available in the OSF repository, https://osf.io/z37d8/.
