# Supplementary material for: Effectiveness of stress arousal reappraisal and stress-is-enhancing mindset interventions on task performance outcomes: a meta-analysis of randomized controlled trials
Source: Sci Rep. 2024 Apr 4;14:7923. doi: 10.1038/s41598-024-58408-w (PMC10994935; doi:10.1038/s41598-024-58408-w)
Supplement: Supplementary file 1 — Supplementary Information. [file 41598_2024_58408_MOESM1_ESM.pdf]

Effectiveness of stress arousal reappraisal and stress-is-enhancing mindset interventions on task performance outcomes: a meta-analysis of randomized controlled trials

Michel Bosshard, Patrick Gomez

Supplementary Material

Supplementary Table S1. PRISMA 2020 Checklist.

Section and Topic	Item #	Checklist item	Location where item is reported
**TITLE**			
Title	1	Identify the report as a systematic review.	Page 1
**ABSTRACT**			
Abstract	2	See the PRISMA 2020 for Abstracts checklist.	Page 2
**INTRODUCTION**			
Rationale	3	Describe the rationale for the review in the context of existing knowledge.	Page 8
Objectives	4	Provide an explicit statement of the objective(s) or question(s) the review addresses.	Page 8
**METHODS**			
Eligibility criteria	5	Specify the inclusion and exclusion criteria for the review and how studies were grouped for the syntheses.	Page 9
Information sources	6	Specify all databases, registers, websites, organisations, reference lists and other sources searched or consulted to identify studies. Specify the date when each source was last searched or consulted.	Pages 9-10
Search strategy	7	Present the full search strategies for all databases, registers and websites, including any filters and limits used.	Supplementary Table S2
Selection process	8	Specify the methods used to decide whether a study met the inclusion criteria of the review, including how many reviewers screened each record and each report retrieved, whether they worked independently, and if applicable, details of automation tools used in the process.	Page 10
Data collection process	9	Specify the methods used to collect data from reports, including how many reviewers collected data from each report, whether they worked independently, any processes for obtaining or confirming data from study investigators, and if applicable, details of automation tools used in the process.	Pages 10-11
Data items	10a	List and define all outcomes for which data were sought. Specify whether all results that were compatible with each outcome domain in each study were sought (e.g. for all measures, time points, analyses), and if not, the methods used to decide which results to collect.	Pages 10-13
	10b	List and define all other variables for which data were sought (e.g. participant and intervention characteristics, funding sources). Describe any assumptions made about any missing or unclear information.	Pages 10-13
Study risk of bias assessment	11	Specify the methods used to assess risk of bias in the included studies, including details of the tool(s) used, how many reviewers assessed each study and whether they worked independently, and if applicable, details of automation tools used in the process.	Supplementary Table S3 and Note
Effect measures	12	Specify for each outcome the effect measure(s) (e.g. risk ratio, mean difference) used in the synthesis or presentation of results.	Page 11
Synthesis methods	13a	Describe the processes used to decide which studies were eligible for each synthesis (e.g. tabulating the study intervention characteristics and comparing against the planned groups for each synthesis (item #5)).	Pages 11-13
	13b	Describe any methods required to prepare the data for presentation or synthesis, such as handling of missing summary statistics, or data conversions.	Pages 11-13
	13c	Describe any methods used to tabulate or visually display results of individual studies and syntheses.	Pages 11

Section and Topic	Item #	Checklist item	Location where item is reported
	13d	Describe any methods used to synthesize results and provide a rationale for the choice(s). If meta-analysis was performed, describe the model(s), method(s) to identify the presence and extent of statistical heterogeneity, and software package(s) used.	Page 11
	13e	Describe any methods used to explore possible causes of heterogeneity among study results (e.g. subgroup analysis, meta-regression).	Pages 13-14
	13f	Describe any sensitivity analyses conducted to assess robustness of the synthesized results.	-
Reporting bias assessment	14	Describe any methods used to assess risk of bias due to missing results in a synthesis (arising from reporting biases).	Page 13
Certainty assessment	15	Describe any methods used to assess certainty (or confidence) in the body of evidence for an outcome.	13
**RESULTS**			
Study selection	16a	Describe the results of the search and selection process, from the number of records identified in the search to the number of studies included in the review, ideally using a flow diagram.	15
	16b	Cite studies that might appear to meet the inclusion criteria, but which were excluded, and explain why they were excluded.	15
Study characteristics	17	Cite each included study and present its characteristics.	Table 1
Risk of bias in studies	18	Present assessments of risk of bias for each included study.	Table 1, Supplementary Table S3
Results of individual studies	19	For all outcomes, present, for each study: (a) summary statistics for each group (where appropriate) and (b) an effect estimate and its precision (e.g. confidence/credible interval), ideally using structured tables or plots.	Figure 2
Results of syntheses	20a	For each synthesis, briefly summarise the characteristics and risk of bias among contributing studies.	Pages 15-16
	20b	Present results of all statistical syntheses conducted. If meta-analysis was done, present for each the summary estimate and its precision (e.g. confidence/credible interval) and measures of statistical heterogeneity. If comparing groups, describe the direction of the effect.	Figure 2
	20c	Present results of all investigations of possible causes of heterogeneity among study results.	Pages 19-20
	20d	Present results of all sensitivity analyses conducted to assess the robustness of the synthesized results.	-
Reporting biases	21	Present assessments of risk of bias due to missing results (arising from reporting biases) for each synthesis assessed.	Page 21
Certainty of evidence	22	Present assessments of certainty (or confidence) in the body of evidence for each outcome assessed.	Pages 16-22
**DISCUSSION**			
Discussion	23a	Provide a general interpretation of the results in the context of other evidence.	Pages 22-25
	23b	Discuss any limitations of the evidence included in the review.	Pages 25-26
	23c	Discuss any limitations of the review processes used.	Pages 25-26
	23d	Discuss implications of the results for practice, policy, and future research.	Page 27

Section and Topic	Item #	Checklist item	Location where item is reported
**OTHER INFORMATION**			
Registration and protocol	24a	Provide registration information for the review, including register name and registration number, or state that the review was not registered.	Page 8
	24b	Indicate where the review protocol can be accessed, or state that a protocol was not prepared.	Page 8
	24c	Describe and explain any amendments to information provided at registration or in the protocol.	
Support	25	Describe sources of financial or non-financial support for the review, and the role of the funders or sponsors in the review.	-
Competing interests	26	Declare any competing interests of review authors.	Page 34
Availability of data, code and other materials	27	Report which of the following are publicly available and where they can be found: template data collection forms; data extracted from included studies; data used for all analyses; analytic code; any other materials used in the review.	Page 28

Supplementary Table S2. Search Strings.

**Database**	**Search string**
Psycinfo (Ovid)	((stress* adj3 reapprais*) or (stress* adj3 refram*) or (stress* adj3 reinterpret*) or (arousal adj3 reapprais*) or (arousal adj3 refram*) or (arousal adj3 reinterpret*) or (anxi* adj3 reapprais*) or (anxi* adj3 refram*) or (anxi* adj3 reinterpret*) or (stress* adj5 mindset*)).ti,ab,id
MEDLINE (Ovid)	((stress* adj3 reapprais*) or (stress* adj3 refram*) or (stress* adj3 reinterpret*) or (arousal adj3 reapprais*) or (arousal adj3 refram*) or (arousal adj3 reinterpret*) or (anxi* adj3 reapprais*) or (anxi* adj3 refram*) or (anxi* adj3 reinterpret*) or (stress* adj5 mindset*)).ti,ab,id
ERIC (Ovid)	((stress* adj3 reapprais*) or (stress* adj3 refram*) or (stress* adj3 reinterpret*) or (arousal adj3 reapprais*) or (arousal adj3 refram*) or (arousal adj3 reinterpret*) or (anxi* adj3 reapprais*) or (anxi* adj3 refram*) or (anxi* adj3 reinterpret*) or (stress* adj5 mindset*)).ti,ab,id
Scopus	TITLE-ABS-KEY (((stress* W/3 reapprais*) OR (stress* W/3 refram*) OR (stress* W/3 reinterpret*) OR (arousal W/3 reapprais*) OR (arousal W/3 refram*) OR (arousal W/3 reinterpret*) OR (anxi* W/3 reapprais*) OR (anxi* W/3 refram*) OR (anxi* W/3 reinterpret*) OR (stress* W/5 mindset*)))
Web of Science	TS=(((stress* NEAR/3 reapprais*) or (stress* NEAR/3 refram*) or (stress* NEAR/3 reinterpret*) or (arousal NEAR/3 reapprais*) or (arousal NEAR/3 refram*) or (arousal NEAR/3 reinterpret*) or (anxi* NEAR/3 reapprais*) or (anxi* NEAR/3 refram*) or (anxi* NEAR/3 reinterpret*) or (stress* NEAR/5 mindset*)))
ProQuest Dissertations & Thesis	abstract(((stress* NEAR/3 reapprais*) OR (stress* NEAR/3 refram*) OR (stress* NEAR/3 reinterpret*) OR (arousal NEAR/3 reapprais*) OR (arousal NEAR/3 refram*) OR (arousal NEAR/3 reinterpret*) OR (anxi* NEAR/3 reapprais*) OR (anxi* NEAR/3 refram*) OR (anxi* NEAR/3 reinterpret*) OR (stress* NEAR/5 mindset*))) OR title(((stress* NEAR/3 reapprais*) OR (stress* NEAR/3 refram*) OR (stress* NEAR/3 reinterpret*) OR (arousal NEAR/3 reapprais*) OR (arousal NEAR/3 refram*) OR (arousal NEAR/3 reinterpret*) OR (anxi* NEAR/3 reapprais*) OR (anxi* NEAR/3 refram*) OR (anxi* NEAR/3 reinterpret*) OR (stress* NEAR/5 mindset*)))
Cochrane	((stress* NEAR/3 reapprais*) or (stress* NEAR/3 refram*) or (stress* NEAR/3 reinterpret*) or (arousal NEAR/3 reapprais*) or (arousal NEAR/3 refram*) or (arousal NEAR/3 reinterpret*) or (anxi* NEAR/3 reapprais*) or (anxi* NEAR/3 refram*) or (anxi* NEAR/3 reinterpret*) or (stress* NEAR/5 mindset*))

Supplementary Table S3. Quality assessment scores.

Study	Item 1	Item 2	Item 3	Item 4	Total score
Akinola et al. 2016	1	1	1	1	4
Baynard-Montague & James 2023	1	1	1	1	4
Beltzer et al. 2014	1	1	1	1	4
Brady et al. 2018	0	1	1	1	3
Chalmers 2018	0	1	1	0	2
Crum et al. 2017	1	1	1	1	4
Crum et al. 2018	1	1	1	1	4
Erazo 2017	0	1	1	0	2
Ganley et al. 2021	0	1	1	1	3
Garcia 1982	0	1	0	0	1
Goyer et al. 2022	0	1	1	1	3
Griffin & Howard 2021	1	1	1	1	4
Gurera & Isaacowitz 2022	0	1	1	1	3
Hangen et al. 2019	1	1	1	1	4
Jacobs 2013	0	1	1	0	2
Jacquart et al. 2020	0	1	1	1	3
Jamieson et al. 2010	0	1	1	1	3
Jamieson et al. 2016	1	1	1	1	4
Jamieson et al. 2022	1	1	1	1	4
John-Henderson et al. 2015	0	1	1	1	3
Johns et al. 2008 (study 3)	0	1	1	1	3
Johns et al. 2008 (study 4)	0	1	1	1	3
Keech et al. 2021	1	1	1	1	4
Mesghina et al. 2021	1	1	1	1	4
Mesghina et al. 2022 (study 1)	1	1	1	1	4
Mesghina et al. 2022 (study 2)	1	1	1	1	4
Moore et al. 2015	0	1	1	1	3
Ott 2017	1	1	1	0	3
Oveis et al. 2020	1	1	1	1	4
Reza et al. 2023	0	1	1	1	3
Rozek et al. 2019	0	1	1	1	3
Sammy et al. 2017	1	1	1	1	4
Taber 2021	1	1	1	0	3
Yeager et al. 2022	0	1	1	1	3

Supplementary Table S3 (Continued).

Study	Item 1	Item 2	Item 3	Item 4	Total score
Zhu 2022	0	1	1	0	2

Supplementary Note 1. Quality assessment items**.**

1. Did the study use an adequate sample size according to an a-priori power analysis or was a genuine reason provided for the sample size? (yes=1, no=0)
2. Were the interventions and control conditions sufficiently described to allow replication? (yes=1, no=0)
3. Were relevant covariates considered in the analysis? (yes=1, no=0)
4. Was the article peer-reviewed? (yes=1, no=0)

Supplementary Table S4. Results for multilevel meta-analysis.

d	**95% CI**	***p***	τ^2_{level2}	τ^2_{level3}	I^2_{level2}	I^2_{level3}
0.23	0.15-0.31	< .001	0.000	0.036	0.0%	51.14%

Note. Level 2 represents within cluster heterogeneity; level 3 represents between cluster (between-study) heterogeneity.

Supplementary Table S5. Results for meta-analysis with robust variance estimates and sensitivity analysis for varying ρ.

	**rho = 0.2**	**rho = 0.4**	**rho = 0.6**	**rho = 0.8**
d	0.23	0.23	0.23	0.23
**95% CI**	0.15-0.32	0.15-0.32	0.15-0.32	0.14-0.32

Supplementary Note 2. Effect size aggregation

Seven studies ([16,28,35,37,91], study 1 & 2 of [68]) reported two different performance outcomes, one study [47] reported three different performance outcomes, and one study reported 4 different performance outcomes[26]. We averaged the different outcomes to produce a single effect size per study. Three studies [34,74,88] reported two different performance outcomes and compared the interventional group to two different control groups (e.g., neutral and SID mindset). In this case, we first calculated the four effect sizes (2 outcomes x 2 comparisons) and then averaged the effect size across the different outcomes for each control group. Therefore, two effect sizes were considered for each of these three studies. Four studies [12,29,30,36] reported separate results for two subgroups, which were treated as independent studies. One of these studies [30] reported two effect sizes for each subgroup, which were averaged to one effect size per subgroup. One study included a neutral and an active control group [51], and another study included a pure SAR and a mixed SAR intervention [57]. For each of the two studies, two effect sizes were considered. For the remaining studies, only one effect size per study was considered ([11, ,14, ,31-33,38,39,49,50,67,69,70,81,89,90], study 3 & 4 in [36]). After aggregation, 44 effect sizes were included in the meta-analysis.
